# Conservative Treatment of Neonatal Brachial Plexus Palsy: A Narrative Review

**DOI:** 10.3390/jcm13247826

**Published:** 2024-12-21

**Authors:** Valentina Boetto, Anna Markova, Federica Malgrati, Isabel Bongiovanni, Anna Bassetto, Chiara Pavese, Antonio Nardone, Giuseppe Massazza, Gabriele Colò, Paolo Titolo

**Affiliations:** 1 Department of Clinical-Surgical, Diagnostic and Pediatric Sciences, University of Pavia, 27100 Pavia, Italy; valentina.boetto01@universitadipavia.it (V.B.); antonio.nardone@icsmaugeri.it (A.N.); 2 Istituti Clinici Scientifici Maugeri IRCCS, Neurorehabilitation and Spinal Unit of Pavia Institute, 27100 Pavia, Italy; 3 Division of Physical and Rehabilitation Medicine, Department of Surgical Sciences, University of Turin, 10126 Turin, Italy; 4 Department of Orthopaedics and Traumatology, Regional Center for Joint Arthroplasty, ASO Alessandria, Via Venezia 16, 16121 Alessandria, Italy; 5 Unit of Hand Surgery, Microsurgery and Reconstructive, Department of Orthopaedics and Traumatology, CTO Hospital, 10126 Turin, Italy

**Keywords:** neonatal brachial plexus palsy, brachial plexus injury, rehabilitation, conservative treatment, upper extremity, obstetrics, children

## Abstract

Neonatal brachial plexus palsy (NBPP) is a flaccid paralysis of the upper limbs that occurs in about 0.4 percent of live births. This condition can produce permanent disabilities; to date, there is no consensus on protocols to be applied for the rehabilitation of children with this condition. The aim of this article is to provide a concise overview of conservative treatment beyond traditional physical therapy for the management of the child with NBPP and to offer a number of useful options for creating the most comprehensive and functional rehabilitation treatment possible. We conducted a narrative review after analyzing articles from the past 50 years on PubMed, Cochrane Library, Scopus, and Web of Science with the following search string [(“neonatal brachial plexus palsy” OR “obstetric brachial plexus palsy” OR “birth brachial plexus palsy”) AND (“rehabilitation” OR “physiotherapy” OR “conservative treatment”)]. We identified a potential of 1275 articles, but only 11 were exclusively about conservative approaches. The most represented rehabilitation approaches in the literature were botulinum toxin, constraint-induced movement therapy (CIMT), virtual reality, neuromuscular electrical stimulation, and kinesiotaping. In conclusion, the various rehabilitation approaches for NBPP are promising, but none can be considered the best option when used alone. In light of the current evidence, a multimodal approach is needed.

## 1. Introduction

Neonatal brachial plexus palsy (NBPP) is a flaccid paralysis of the upper extremity occurring during childbirth [1,2]. The incidence of NBPP varies in different parts of the world due to differences in social standards, with more institutional deliveries and improved obstetric care and management.

The incidence of NBPP in the world is approximately between 0.1% and 0.7% live births annually; it varies in different parts of the world due to differences in social standards, with more institutional deliveries and improved obstetric care and management. In North America it corresponds to 0.4% live births in the USA, 0.124% live births in Canada. In India, the incidence is approximately 0.4 to 2.5 per 1000 live births [2,3,4,5].

The first clinical description of NBPP was by Smellie in 1760. In the late 1800s, different types of NBPP were defined. In particular, in 1873 Wilhelm H. Erb described injury of the upper nerve of the trunk to the nerve roots C5 and C6, while in 1885 Augusta Klumpke described a lesion of the lower trunk involving the nerve roots C8 and T1. Later, in the early 1900s, paralysis of all nerve roots from C5 to T1 was described.

**Figure 1:**
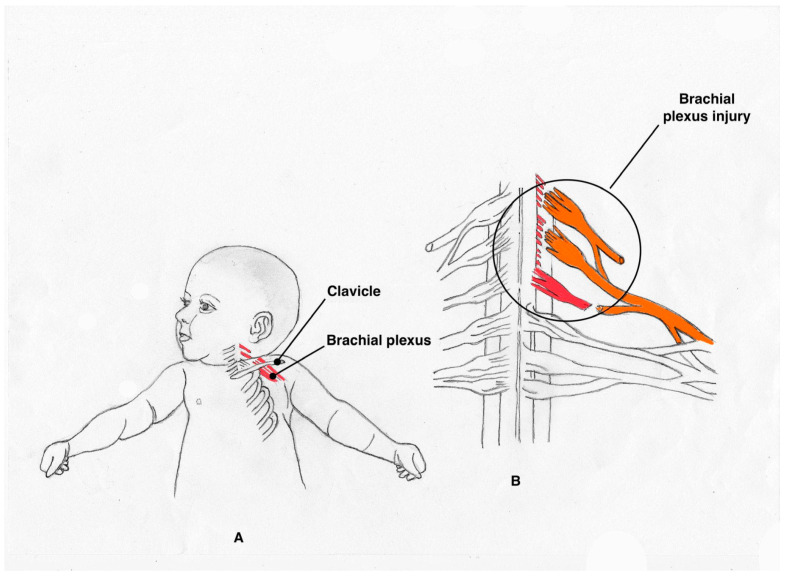
A schematic drawing by Dr. Coló of the brachial plexus palsy: (**A**) relation between plexus and clavicle, (**B**) plexus injury.

In most cases, this injury stems from a compressive or traction trauma exerted by nearby structures (ribs, vertebrae, or muscles) on the brachial plexus (Figure 1). The most frequent cause of NBPP is unilateral traction damage caused by the forces needed to release the front or rear aspect of the shoulder of the unborn child locked behind the pubic symphysis or the sacral promontory of the mother; this is also known as shoulder dystocia. Other less frequent causes are hemangioma, tumors, inflammatory diseases, and diagnostic or therapeutic procedures [6]. Risk factors for NBPP include gestational diabetes with birth weight above the 90th percentile (4500 to 5000 g), prolonged labor, induction of childbirth, and use of mechanical assistance (vacuum, forceps) [6,7,8,9,10,11,12,13].

NBPP can cause upper extremity deficit in infants ranging from mild injuries, which resolve completely, to severe and permanent disabilities [14]. In 1987, Narakas classified infants with NBBP into four groups: (I) upper Erb, (II) extended Erb, (III) total palsy, and (IV) Horner’s total palsy; this classification is still widely used today in clinical practice as it provides a general view of the expected prognosis soon after the birth of the affected infant.

Group I is characterized by lesions to the nerve roots of C5 and C6, manifesting as paresis/paralysis of the deltoid and biceps while the function of the extensor muscles in the upper limb is preserved. Group II in addition to the above lesions also involves C7, with paresis/paralysis not only of the deltoid and biceps, but also of triceps and wrist extensors, while the long flexors and intrinsic muscles of the hand are preserved. In group III, all the nerve roots (C5, C6, C7, C8, and T1) of the brachial plexus are lesioned with paresis/paralysis of the muscles of the entire arm. Group IV in addition to paresis/paralysis of the muscles of the entire arm, similar to group III, also presents Horner syndrome, i.e., ptosis, miosis, and anhidrosis of the face ipsilateral to the lesion, due to a sympathetic lesion. This implies a lesion of all nerve roots of the brachial plexus with injury proximal to the lower nerve roots. At the epidemiological level, the most frequent lesions (73%) are those involving the upper brachial plexus (C5–C7) known as Erb group II lesions [15].

When this classification system is used between two and four weeks after birth, it facilitates the determination of the extent of injury to guide the prognosis and subsequent management. The estimated prognosis for groups I and II is favorable, with spontaneous recovery possible in about 80% of patients in group I and 60% in group II. For groups III and IV, the prognosis worsens drastically with spontaneous recovery possible in only about 40% of patients in group III, while it is almost absent in group IV [16,17,18,19,20].

Over the years, the classification has been refined, particularly concerning the prognostic effect of early recovery of wrist extension against gravity before two months of age, dividing group II into two subcategories: IIA, Extended Erb’s with early recovery of wrist extension; and IIB, Extended Erb’s with no early recovery of wrist extension [21].

In literature, surgical approaches are described and planned according to the type of injury (Narakas group I, II, etc.), while regarding the rehabilitation approach, very few studies take into account the level of injury of the participants and clearly specify this in the study design.

As anticipated by Narakas classification, the most common and significant deformities resulting from NBPP are medial rotation contracture and posterior subluxation and dislocation of the humeral head. Functionally, this condition can cause significant deficits, severely limiting the patient’s ability to perform bimanual tasks and activities of daily living. In the long term, the quality of life of NBPP patients is not significantly affected, but this condition impacts their education, career choices, and work performance. Early guidance and tailored physical and psychosocial support could help prevent or reduce these challenges, improving participation in work and education.

With a rehabilitation program, the main complications of NBPP can be prevented, such as muscle atrophy, the development of imbalance, contracture, formation of bone and muscle deformities, pain, and somatosensory deficits [5].

The choice of whether to proceed with conservative treatment or opt for surgery in a child affected by NBPP is still a matter of debate, and it presupposes early diagnosis and treatment of the patient. According to Narakas classification, group IV, as the one with the more severe lesion, is a candidate for early neurosurgical intervention [15]. For the other groups, there is still no clear evidence.

Making such a decision requires the coordination of a multidisciplinary team composed of a physiatrist, clinical neurophysiologist, orthopedic surgeon, neurosurgeon, occupational therapist, and physical therapists [16].

There is currently no scientific evidence to support a protocol for the conservative management of NBPP, and the available literature on specific rehabilitation techniques has a low level of evidence.

The aim of this article is to provide a concise overview of the conservative treatment, beyond traditional physiotherapy for the management of the child with NBPP, and to offer a range of options that will be useful in creating the most comprehensive and functional rehabilitation treatment possible.

## 2. Search Strategy

We conducted a review of the literature using PubMed, Cochrane Library, Scopus, and Web of Science. The following combination of terms was used: [(“neonatal brachial plexus palsy” OR “obstetric brachial plexus palsy” OR “birth brachial plexus palsy”) AND (“rehabilitation” OR “physiotherapy” OR “conservative treatment”)]. 

We applied the following criteria to include or exclude studies in the review. Inclusion criteria: (1) population: patients with NBPP in an age-range from 1 month to 18 years; (2) description: communication of rehabilitation protocols or management interventions for NBPP; (3) study design: interventional studies, observational studies, case series, and case reports; (4) language: English; and (5) publication time: from January 1974 to 30 March 2024. Exclusion criteria: (1) population: patients who have undergone surgery before treatment; patients with other musculoskeletal or neurological conditions as control group; and study design: literature reviews, meta-analyses, and conference proceedings, full text not available.

## 3. Results

We identified 1275 potentially relevant articles. Based on the screening of the title and abstract, 1186 articles were excluded because they did not address a rehabilitation technique. Of the remaining 89 articles, after full-text analysis, only 11 were about only conservative approaches. We did not consider articles concerning patients who had surgery before treatment or patients with other musculoskeletal or neurological conditions. The 11 articles we identified as the most relevant included patients with NBPP in an age-range from 1 month to 18 years.

Table 1 summarizes the main information of the 11 studies included in this review. 

The studies investigated various treatments: botulinum toxin (n = 2) [22,23], constraint-induced movement therapy (n = 2) [24,25], virtual reality (n = 3) [26,27,28], neuromuscular electrical stimulation (n = 1) [29], and kinesiotaping (n = 3) [30,31,32]. The articles selected were extremely heterogeneous in terms of study design and level of evidence (1 case-control [31], 1 cohort studies [23], 6 randomized controlled trials (RCTs) [22,24,26,28,29,32], 1 quasi-randomized study [30], 1 randomized pilot study [27], and 1 randomized crossover trial [25]). The ages of the patients in the different studies varied greatly, from one-month-old infants to 10 years old.

We report below the findings about the specific treatments.

### 3.1. Botulinum Toxin

Botulinum toxin (BTX) is routinely used for medical treatment in conditions ranging from classic indications such as muscle spasticity to dystonia and the management of wrinkles. Treatment with BTX is safe and effective in children with NBPP as demonstrated by a recent meta-analysis by Chen et al. [33]. As in adults, also in children, ultrasound-guided injections are useful to increase the accuracy of the procedure [34].

In the last five years, it has been shown that early treatment with BTX (under 2 years of age) has positive effects on the development and growth of the child [22,35].

One of the latest studies showed that use of a single injection of Onabotulinum toxin type-A (OBTT-A) in the first 6 months of age was an early and effective treatment that could avoid bone deformity and the consequential surgical treatment (Table 1) [23]. However, it is very difficult to prove that better function after a plexus injury depends on an early treatment as the spontaneous healing is not the same for every child.

Recent studies with magnetic resonance imaging have shown an improvement in the plasticity of the corticospinal tract in children with NBPP after treatment with BTX type-A (BTX-A). An increased plasticity of the corticospinal tract could have a positive effect on the prognosis of peripheral injuries and could be a rehabilitation strategy for a better motor outcome in NBPP [36].

### 3.2. Constraint-Induced Movement Therapy

Constraint-induced movement therapy (CIMT) is among the most promising rehabilitation treatments improving upper extremity function in individuals with neurological dysfunctions [26]. This technique consists in constraining the use of non-affected limb in association with intensive task-related training of the affected limb. In pediatric patients, a modified CIMT (mCIMT) is preferred: to increase its feasibility, the duration of restriction and intensity are reduced, while the constraint methods do not include casting. Despite extensive literature focused on the use of mCIMT in cerebral palsy patients, relatively few studies address its use in children’s peripheral neurological diseases such as NBPP [37]. However, over the years, the number and quality of studies in support of this method in NBPP gradually increased, starting from a few case reports [5,38] up to the latest RCTs and reviews [24,25,37,39].

mCIMT can be associated with other complementary rehabilitative techniques, such as neuromuscular electrical stimulation (NmES) of agonist muscles to enhance active motion as shown in the study of Berggren et al., and it can be associated with BTX-A in antagonist muscles to reduce muscle imbalance and co-contractions [40].

To date, there is no standard protocol on the duration and mode of treatment with mCIMT, but all studies have shown an increase in mobility, functional capacity, speed, range of motion (ROM), hand manipulation ability, and predisposition to use the injured limb. mCIMT also seems to counteract the phenomenon known as “learned non-use” by promoting greater involvement of the affected limb in activities of daily living (ADLs) [41]. In this context, Werner et al. [25] found mCIMT to be superior to conventional therapy in terms of the performance of bimanual activity, a prerequisite for the execution of several ADLs involving the upper limbs.

### 3.3. Virtual Reality

Increasingly, technological rehabilitation techniques are used in the treatment of neurological injuries, and they are also emerging in the treatment of NBPP [26]. A promising study concluded that the virtual reality (VR) program is significantly more effective than conventional physiotherapy in improving upper limb function in patients with NBPP. A VR system allows the therapist to adapt the level of difficulty and motor intensity to match the child’s clinical condition, thus creating goal-oriented and task-specific training programs. In addition, therapists may also monitor the results of the child’s movements and correct them if inappropriate. VR for the treatment of NBPP is therefore an emerging and fascinating therapeutic possibility; however, it remains a prerogative mostly for children from school age onwards, as it needs good compliance in performing the required commands and a good level of attention during treatment [27]. In addition, few centers possess the machinery required to implement it [26,27,28]. Although recent studies have large sample sizes, there is still not enough scientific evidence in the literature to support these new techniques [42].

### 3.4. Neuromuscular Electrical Stimulation

Neuromuscular electrical stimulation (NmES) involves the application of electrodes attached to a device that delivers electrical current to a partially or completely denervated muscle to promote functional recovery [43]. NmES has been used by therapists in the rehabilitation of various pediatric central neurological disorders, including stroke, cerebral palsy, and spinal cord injury, with remarkable demonstrable success [44]. Although there is no standardized use of NmES in patients with NBPP, evidence of its efficacy in the treatment of NBPP has been demonstrated, particularly regarding the increase in ROM. The study by Elnaggar [29] provides evidence that NmES delivered during a multi-component intervention aimed to load the affected arm and perform functional exercises may improve shoulder function and total bone mineral density of the humerus in children. In conclusion, NmES shows encouraging results, is well tolerated by children, and does not require expensive equipment; therefore, it should be included in rehabilitation programs of NBPP. However, the use of this treatment is still rare [45].

### 3.5. Kinesiotaping

In the treatment of children, the use of severe bracing to stretch shoulder internal rotators is not recommended because orthoses are not well tolerated due to fear and discomfort. It has been demonstrated that the use of kinesiotaping can effectively replace the use of splints, with little trauma to the patient and a minimal cost for the family [46].

Recent studies showed that kinesiotaping in association with selected physical therapy improves active movements and functional activities in children with NBPP [31]. This improvement has been assessed not only from a functional viewpoint, with standardized scales such as the Active Movement Scale [47] and the Modified Mallet Classification, but also objectively, using EMG devices to show the different percentages of muscle degeneration after physical therapy and kinesiotaping [30]. Furthermore, kinesiotaping proved so promising that some authors tried to pinpoint the most useful place in specific conditions. For example, it has been demonstrated that the most effective configuration for scapular stabilization in children with NBPP is on the middle and lower trapezius. Russo et al. reported that this position of taping improved posture by decreasing scapular winging and glenohumeral internal rotation. In order to apply the tape, participants retracted their scapulae toward the spine, and the therapist manually augmented this scapular motion while applying the tape. Conversely, rhomboids taping should be avoided as no benefit was found [48].

In conclusion, kinesiotaping is a method with good patient compliance and low cost; this technique, together with conventional treatment, may be beneficial in preventing limitations and in contributing to functional development [32].

## 4. Discussion

All studies show that the vast majority of obstetrical brachial plexus palsies resolve spontaneously, but in cases where this does not occur, the development of contractures and deformities lead to major functional deficits. There is consensus regarding the use of physical exercises for the treatment of children with NBPP that include a range of functional, daily living, and play activities similar to those routinely practiced every day [19]. All authors agree that it is essential to involve the parents in the rehabilitation program [49]. As soon as the child shows intentional voluntary control, it is important to stimulate the affected limb to avoid developmental apraxia: encouraging children to perform bimanual activities is an important strategy for this purpose. In addition, it is important to avoid muscle contractures and to train parents to perform passive mobilization exercises several times a day [17].

There are several approaches used as complementary means and/or techniques to the conservative treatment of NBPP, including the use of botulinum toxin (BTX) injection, modified constraint-induced movement therapy (CIMT), virtual reality therapy, electrostimulation, and kinesiotaping [14].

As concerns botulinum toxin, therapeutic benefits were found after a single injection of BTX such as in the study by Morscher et al. [23]. However, the use of botulinum toxin is expensive and it requires highly specialized healthcare staff. Moreover, there are possible adverse effects, such as post-infiltration hematoma, muscle weakness, dysphagia, and aspiration pneumonia; anyway, they occur above all when the recommended doses have not been respected.

CIMT is an extremely valid method, but it is rarely used for the difficult organization and management of treatment. The proposal to restrict the healthy limb to require the use of the affected limb is often not well accepted by families. There is no uniformity in the protocols and often the contentions are made in the most disparate ways, the more rigid and not removable, the less the tolerance of patients, and likewise, the acceptance of the therapeutic proposal by families. For its implementation, this technique requires an optimal relationship of communication and trust between the health staff and the family, a high degree of caregiver involvement, and positive management of the possible initial frustration that the child develops with compulsion.

Virtual reality is the therapeutic application of new technological frontiers. All the authors of the examined texts agree on the superiority of virtual reality compared to conventional treatment in terms of upper limb functionality, quality of life, and ability to perform daily activities. No side effects were reported in the studies. However, it remains an uncommon technology because it is expensive and difficult to find and also requires more attention from the patient. The challenge in the coming years will be to succeed in spreading these new technologies more widely so that a larger sample of people can be used to assess their real effectiveness.

The technology required for NMES is widely used. In the adult patient it is used with excellent results to the point that the most modern orthoses integrated it to improve the functionality. The procedure can sometimes be painful, so it is not always tolerated by patients. Moreover, there is no uniformity in the protocols, as impulse, duration, and timing of treatment have large variability. Nowadays, it is unclear what is the best setting to perform NMES

Kinesiotaping is one of the conservative treatments that can be used right from the start, after making the diagnosis. In ElKhatib RS’s study [30], it was used as an adjunct to a physical therapy program and showed better, earlier, and smoother recovery of the affected arm than using the therapy program alone. Moreover, it is a cheap technique and well tolerated by patients [50]. Only one study [31] mentioned skin sensitivity (skin hyperemia or small spots) as an exclusion criteria, and no participants had adverse effects.

This literature review shows that the treatment offered to children affected by NBPP is heterogeneous. Across studies, the sample size was extremely small in some cases (from a minimum of 12 patients to 90 patients in the largest study). In most of the studies examined, there was no control group. Few studies included a follow-up in their description and, when available, the duration was shorter than 1 year. This precluded the possibility of evaluating the long-term effects of the proposed treatments.

The heterogeneity of the studies regarded not only the different types of treatment, but also the different modes for initiating children in the therapeutic process. By analyzing the data, it was not possible to identify the most suitable age to start the treatment. The age of the patients considered by the different studies varied widely, from babies as young as one month old to children almost 10 years old.

Additionally, the different types of lesions leading to NBPP were compared and treated across the various studies in the same way in spite of being lesions with different deficits, i.e., no cluster analysis was possible. In this review we excluded all studies that had patients who previously underwent surgery for NBPP in their sample. However, this does not allow us to determine whether conservative treatment alone is sufficient or if surgery will be necessary in the therapeutic pathway for these children. With respect to the duration of treatment, the studies exhibited significant heterogeneity, ranging from a minimum of one single session [23] to a maximum of 4 months [25]. Additionally, concerning the goals to be achieved, we observed a considerable diversity. In some studies, the primary aim was to avoid surgery, while in many others, the goals focused on improving ADLs and promoting voluntary activation of the affected limb. Given the disparity in objectives, it was challenging to compare the outcomes across studies due to the extensive diversity in the assessment scales employed.

The outcome of all these discrepancies is that it was impossible to determine what the best therapeutic approach may be. Nevertheless, considering the number and sample sizes of the studies carried out investigating the use of BTX and CIMT, we found BTX and CIMT as potentially being the therapies with stronger evidence. Consequently, the most recent and methodologically robust studies focused on assessing the effects and applicability of these two approaches in treating children affected by NBPP.

## 5. Conclusions

This review highlights both the variability of potential treatments in addition to conservative physiotherapy and the lack of conclusive scientific evidence to support them. The use of botulinum toxin is expensive, requires highly specialized healthcare staff, careful patient selection, and long treatment and follow-up periods. CIMT is an extremely valuable method but is often not very well accepted by patients and families. Virtual reality offers new perspectives, but it is an expensive and still uncommon technology and more attention from the patient is needed. The technology required for NMES, on the other hand, is inexpensive and widely used but the procedure can sometimes be painful, so it is not always tolerated. However, Kinesiotaping is extremely cheap, easy to obtain and use, and very well tolerated by patients and relatives.

All rehabilitation protocols described are experimental and show benefit when integrated with additional clinical treatment modalities; none of them, if taken alone, could be considered the best choice.

Future studies are necessary to optimize the rehabilitation approach and refine the protocols according to patient age and severity of paralysis, taking into account the need for adequate sample size. We strongly recommend that future research should also focus on standardizing the duration of treatment and homogenizing the outcome assessment tools. Only with this evidence will we be able to identify the optimal treatment option to improve the quality of life of children with NBPP.

## Figures and Tables

**Table 1:** Summary of findings: NBPP treatments.

Author and Year	Design	Sample & Mean Age	Type of Treatment	Aims	Timing	Study Group Treatment	Control Group Treatment	Outcome	Follow Up
Pons et al. 2019 [22]	Prospective Multicentre RCT	62 0.875 years old	BTX (onabotulinumtoxinA)	Evaluate BTX effectiveness in limiting posterior subluxation of the glenohumeral joint. Secondary aims include assessing glenoid retroversion, three-dimensional deformity, shoulder ROM, and upper limb function	Single-session injection; physiotherapy sessions twice a week (both groups)	BTX (8UI/kg) injected into the pectoralis major, subscapularis and teres major/latissimus dorsi muscles. Ultrasound guidance	Sham procedure mimicking BTX without injection.	Strong evidence of BTX effectiveness	until 18 months of age
Morscher et al. 2020 [23]	Retrospective Observational Cohort Study	12 <0.5 years old	BTX (onabotulinumtoxinA)	Present experience with BTX injections to enhance elbow flexion. Diagnostically, a post-injection Toronto Test Score < 2.0 at 8 months indicated surgery	Single injection to triceps muscle	BTX (10 to 30 units) injected into the triceps. EMG or EMS	Not specified	Increased Toronto elbow flexion scores in 10 of 12 infants. 4 of 9 infants achieved full elbow flexion.	Monthly for the first year
Eren et al. 2020 [24]	Prospective Single-blind RCT	39 4.7 years old	mCIMT	Compare mCIMT and conventional therapy in NBPP infants (evaluating upper limb function).	1 h/day, 14 consecutive days; hospital setting	Study group was hospitalized for 14 days. Restriction was attained by dressing a custom-made static upper limb orthosis, 6 h/day (2 h on- 1 h off for 3 cycles)	1 h/day of physiotherapy for 14 consecutive days. Home exercise program in both groups	mCIMT group improved more than control group in active ROMs and upper extremity function	After 3 months
Werner et al. 2021 [25]	Randomized Crossover Trial	21 2 years old	mCIMT	Determine if mCIMT is more effective than conservative treatment in NBPP children. (evaluating bimanual activity performance)	16-week crossover trial	3 weeks casting unaffected limb, 5 weeks OT and home programming	1 h of clinic-based intervention and family instruction and 2 h of daily home programming	mCIMT superior for bimanual activity performance	NS
El-Shamy et al. 2017 [26]	RCT	40 6.5 years old	Virtual reality program (associated to arm weight support through Armeo^®^ Spring Pediatric, Hocoma, AG, Switzerland)	Evaluate virtual reality vs. conventional physiotherapy effects	45 min/day, 3 times/week, 12 weeks	Practice with virtual reality with the aid of Armeo^®^ Spring Pediatric	Conventional physiotherapy	Virtual reality more effective than conventional physiotherapy	NS
Yeves-Lite et al. 2020 [27]	Randomized Pilot Study	12 8.5 years old.	Virtual reality MT program	Compare effects of conventional MT and virtual Reality MT on spontaneous use of the affected upper limb	20 min/day, 3 days/week, 4 weeks	The intervention protocol consisted of 6 exercises (3 forearm prono-supination exercises and 3 wrist flexion-extension exercises)	Both groups performed the same exercises in virtual reality or MT environment according to the assignment group	Virtual Reality MT complements conventional MT, increasing independent bimanual tasks and improving quality of life	NS
Alsakhawi and Atya, 2020 [28]	RCT	45 8 years old.	Augmented biofeedback (E-Link Upper Limb Exerciser^TM^)	Evaluate effects of augmented biofeedback vs. traditional physical therapy program	60 min/treatment session, 3 times/week, 6 weeks	Physical therapy in addition to the augmented biofeedback system for 30 min	60 min of traditional physical therapy	Adding augmented biofeedback to physical therapy provided greater improvement in upper limb mobility and functional activities	NS
Elnaggar, 2016 [29]	RCT	42 3.8 years old.	NMES	Evaluate effects of NMES during weight-bearing exercises on shoulder function and bone mineral density	40 min/day, 5 days/week, 12 weeks	Traditional exercise plus neuromuscular electrical stimulation during weight bearing	Traditional exercise program	NMES during weight-bearing exercises may improve shoulder function and total BMD of the humerus in children with NBPP	NS
EIKhatib et al. 2012 [30]	Quasi-randomized Study	30 0.3 years old.	Kinesiotaping	Investigate the effect of kinesiotaping over the deltoid and the forearm on the development of proper upper extremity function	45 min of physical therapy/day, 3 times/week for 3 months; tape applied for 3–5 days, removed for 24 h, then reapplied	Kinesiotaping over the deltoid and the forearm associated to physical therapy program	Physical therapy program only	Kinesiotaping of deltoid and forearm, as an adjunct, improved upper extremity function in children with Erb’s palsy	NS
Kamal-Eldeen and Awooda, 2016 [31]	Case Control	30 1.5 years old.	Kinesiotaping	Assess the effect of Kinesiotape to stimulate the extensor muscles	45 min daily for 15 sessions	Kinesiotaping of the wrist extension muscles + physical therapy program	Physical therapy program only	Statistically significant evidence that Kinesio tape, in addition to selected physical therapy, improved active wrist extension and functional activity	NS
Çekmece et al. 2023 [32]	RCT	90 5 years old.	Kinesiotaping	Investigate the effect of Kinesiotape application associated with exercises	45 min/day, 1 day/week for 3 months; tape applied for study group	Kinesiotaping over the scapula and forearm to increase scapular stabilization and active supination	Physical therapy program only	Statistically significant differences in favour of the study group for external rotation of shoulder and for shoulder flexion and elbow flexion	NS

## Data Availability

Not applicable.

## Abbreviations

AHA	Assisting Hand Assessment;
AMS	Active Movement Scale;
BBT	Box and Blocks Test;
BTX	botulinum toxin;
EMG	Electromyography;
EMS	Electrical Muscle Stimulation;
FU	Follow Up;
MC	Mallet Classification;
mCIMT	modified Constraint-Induced Movement Therapy;
MMC	Modified Mallet Classification;
MT	Mirror therapy;
NBPP	Neonatal Brachial Plexus Palsy;
NMES	Neuromuscular Electrical Stimulation;
NS	Not Specified
OBTT-A	Onabotulinum toxin type A;
OT	occupational therapy;
PMAL-R	Pediatric Motor Activity Log-Revised;
RCT	Randomised Controlled Trial;
ROM	Range of Motion
CHEQ	Children’s Hand-use Experience Questionnaire
